# Towards a pragmatic message on optimizing sun exposure

**DOI:** 10.1093/skinhd/vzag041

**Published:** 2026-05-07

**Authors:** Dimitrios Karponis, Dimitrios Bismpos

**Affiliations:** Norwich Medical School, University of East Anglia, Norwich, UK; Department of Dermatology, Norfolk and Norwich University Hospital, Norwich, UK; Department of Cardiology/Angiology, Marien Hospital Herne, Ruhr University, Herne, Germany

## Abstract

A clear and pragmatic message is needed to guide patients on how to optimize their sun exposure. The power of moderation combined with the antifragility of human skin may provide a useful framework to build on.

How much sunlight should we advise patients to receive daily? Too little sunlight exposure has been associated with higher all-cause mortality, while excessive exposure is linked with an increased risk of skin cancer.^1^ Experts’ opinions vary, and the increasing use of social media and artificial intelligence chatbots heralds the risk of (mis)information overload. In the UK, responsible exposure [such as wearing protective clothing, seeking shade, applying sunscreen and avoiding unprotected exposure at ultraviolet (UV) index ≥3] is advised throughout the months of March to October, especially between 11:00 h and 15:00 h.^2^ Would the same advice apply to people in Peru, Bolivia or other parts of the world where the UV index never falls below 3? Here, the words of sixth-century B.c. Greek philosopher Cleobulus, ‘everything in moderation’, serve as a useful guide.

Sunlight is comprised of three types of radiation: infrared (∼50%), visible (∼42%) and UV (<8%). UV radiation reaching the surface of Earth is a known mutagen and can be subdivided to erythemogenic UVB (280–315 nm) and deeply penetrating UVA (315–400 nm). To defend itself from the carcinogenic and ageing effects of UV radiation, human skin has developed multiple DNA repair mechanisms.^3^ These are subject to saturation, as is the case with intense intermittent exposures leading to sunburn and the use of tanning beds. Sun-protection behaviours reduce workload on these innate mechanisms. At the same time, it is important to maintain adequate levels of vitamin D (a surrogate for sunlight exposure, as UVB promotes vitamin D formation) to avoid rickets, osteopenia or osteoporosis.

Mortality from melanoma is slowly declining in England and other countries, while incidence is increasing, especially for *in situ* melanomas.^4^ Multiple explanations have been offered, including earlier diagnosis due to increased public awareness, availability of targeted therapies and immunotherapy, overdiagnosis of thinner tumours that carry a better prognosis and drifting (‘lowering’) histological diagnostic thresholds on what qualifies as melanoma. However, cardiovascular disease is the leading cause of death worldwide; UVA exposure mobilizes cutaneous nitric oxide to the circulation, reducing blood pressure and probably lowering all-cause mortality through a reduction in cardiovascular deaths.^5^

Thorough sun-protection behaviours, apart from blunting the aforementioned mechanism and to a lesser extent arguably interfering with vitamin D production, are expensive (especially when sunscreen is applied at the scientifically recommended dose of 2 mg cm^−2^ or when UV-tested protecting clothing is used) and laborious (checking the UV index on a smartphone before leaving the house would take some getting used to) and sunscreens are the most common cause of photocontact allergy. Regular vitamin D supplementation may also carry cumulative risks of ingesting processed excipients with unknown long-term health effects.

The knowledge of the benefits of light on the skin in health and disease is not novel. The first Nobel Prize in dermatology was awarded to Niels Finsen in 1903 for the treatment of lupus ­vulgaris using UV radiation.^6^ The immunomodulatory, cytotoxic and antimicrobial effects of phototherapy are widely harnessed to this day to treat a plethora of conditions, including those that are inflammatory (e.g. psoriasis), depositional (e.g. morphoea), photoinduced (e.g. polymorphic light eruption), infective (e.g. onychomycosis), premalignant (e.g. actinic keratoses) and malignant (e.g. basal cell carcinoma), among others.

Hormesis may present a practical approach to capture the benefits of sunlight while minimizing the risks, responsibly. Following Taleb’s framework in *Antifragile*, when systems are challenged with low-dose stressors they become more resilient, or, in the case of complex dynamic systems, they gain from disorder altogether.^7,8^ In the case of sunlight and human skin, regulated unprotected exposures to UV index <3, or as low as possible in cases where the UV index is ≥3, can ‘harden’ the skin and may ‘sharpen’ DNA repair mechanisms. The duration of exposure will vary for each individual according to their circumstances. The key is not to overwhelm DNA repair mechanisms, while noting that occult mutational damage occurs at suberythemal doses of UV radiation.^9^

While this practice may be in contrast to the indoor lifestyle of the twenty-first century, it is in line with the independent evolution of lighter *Homo sapiens* skin at higher latitudes, highlighting the significance of sunlight for human health.^10^ A vitamin D level can be measured to refine one’s practice of sunlight hormesis and rule out insufficiency, but regular monitoring is unlikely to be beneficial or cost-efficient for most people.

Are there any limitations or exceptions to sunlight hormesis? Firstly, the use of tanning beds does not qualify as sunlight hormesis; the stressor is too great for the body to compensate and instead results in pure mutational damage and increases the risk of skin cancer. Hormesis should be avoided in patients with genetic DNA excision repair defects that result in photosensitivity (such as xeroderma pigmentosum, Bloom syndrome and Rothmund–Thomson syndrome), as there is a markedly reduced capacity to repair mutational damage. Another group of patients for whom hormesis may not be appropriate is those most concerned about cosmesis. The most effective way to minimize photoageing is daily sunscreen use (along with all other sun-protection behaviours). However, this cohort must be warned about the risks of UV radiation deficiency and the impending fragility of their skin in an unprotected state.

The subjectivity in ‘moderation’ behind hormesis is not a cause for concern; how could there be a one-size-fits-all ­recommendation for the general population, ignoring the differences in genotype, phenotype, behaviour and environmental exposures? After all, these are the traits that render human skin, the outline of our physical existence and barrier to the world, unique. The ‘good’ and ‘bad’ nature of sunlight can be compared with the duality of light in quantum physics, behaving as both a wave and a particle. In essence, ‘moderation’ pragmatically reinforces the idea that extremes (such as sunburns and complete sun abstinence) should be avoided, while encouraging personalized adherence to guidance from professional bodies, without promoting health anxiety.

The wisdom of the ancient Greeks lives on and is reflected in the dictum of the famous sixteenth-century A.d. Swiss physician–alchemist Paracelsus that ‘the dose makes the poison’. Sunlight is ­neither a villain nor a panacea. An evidence-based, pragmatic and nonjudgemental message is needed to empower our patients to enjoy the sun responsibly.

## Data Availability

No new data were generated. Any references made to other works have been cited appropriately.
